# Long-term outcomes of canaloplasty and phaco-canaloplasty in the treatment of open angle glaucoma: a single-surgeon experience

**DOI:** 10.1007/s10792-024-03174-x

**Published:** 2024-07-07

**Authors:** Daniele Tognetto, Gabriella Cirigliano, Stefano Gouigoux, Alberto Grotto, Pier Luigi Guerin, Leandro Inferrera, Dario Marangoni

**Affiliations:** University Eye Clinic, Department of Medicine, Surgery and Health Sciences, University of Trieste, 34129 Trieste, Italy

**Keywords:** Canaloplasty, Phaco-canaloplasty, Primary open angle glaucoma, Intraocular pressure, Glaucoma medications

## Abstract

**Purpose:**

To evaluate and compare the long-term outcomes of canaloplasty and phaco-canaloplasty in the treatment of open angle glaucoma and assess the prognostic factors associated with surgical outcome.

**Methods:**

A 48-month retrospective analysis was performed on *n* = 133 open angle glaucoma eyes treated with canaloplasty and *n* = 57 open angle glaucoma eyes treated with phaco-canaloplasty by a single surgeon. Surgical success was defined according to six criteria, achieving a target intraocular pressure (IOP) ≤ 21, 18 or 15 mmHg on glaucoma medications (qualified success) or without any further treatment (complete success), including laser therapy or surgery. Kaplan–Meier survival analysis and Cox regression analysis were performed to evaluate surgical success and preoperative factors associated with surgical outcome. Surgical complications in the early postoperative period were compared between canaloplasty and phaco-canaloplasty.

**Results:**

Canaloplasty and phaco-canaloplasty significantly reduced postoperative IOP and number of glaucoma medications (*p* = 0.001 for both). Phaco-canaloplasty showed higher rates of cumulative surgical success over canaloplasty, but only for target IOP ≤ 21 and ≤ 18 (*p* = 0.018 and *p* = 0.011, respectively). A preoperative number of > 4 glaucoma medications predicted surgical failure. Phaco-canaloplasty was associated with a higher rate of IOP peaks in the first month compared with canaloplasty (40.4% vs 12.7%, *p* = 0.000).

**Conclusion:**

Canaloplasty and phaco-canaloplasty demonstrated long-term efficacy in the treatment of open angle glaucoma, with phaco-canaloplasty showing higher rates of surgical success compared to canaloplasty, but not for target IOPs lower than 16 mmHg. Patients on more than 4 preoperative glaucoma medications may not be good candidates for canaloplasty and may benefit from other surgical options.

**Supplementary Information:**

The online version contains supplementary material available at 10.1007/s10792-024-03174-x.

## Introduction

Open angle glaucoma (OAG) is a progressive degenerative optic neuropathy and one of the leading causes of irreversible blindness worldwide [1]. An elevated intraocular pressure (IOP) is the most important risk factor for glaucoma development and progression and surgery represents the ultimate option when IOP cannot be controlled by topical medications or laser procedures [2–7].

Ab-externo canaloplasty (CP) is a non-filtering, non-penetrating surgical procedure which reduces IOP by increasing the humor aqueous flow through the physiological pathway [8]. This procedure involves the dilation of Schlemm’s canal by using a microcatheter and a viscoelastic agent, and placing a tension suture to stretch the trabecular meshwork [9–11]. The rationale behind CP is the anatomical evidence that Schlemm’s canal is significantly narrowed in primary open angle glaucoma (POAG) eyes and 90% of the collector channels are blocked by herniations of the trabecular meshwork [12, 13].

Although the hypotensive efficacy of CP is inferior to filtering surgeries, like trabeculectomy [14–17], CP has the advantage of carrying less postoperative complications, such as bleb leakage, hypotony or endophthalmitis, and therefore to require less intense postoperative surveillance [14–17]. In addition, because it does not rely on the creation of a subconjunctival bleb, CP is a suitable option in patients with chronic conjunctivitis by long-term use of topical antiglaucoma medications, who are at higher risk of bleb failure. CP may be therefore a valid alternative to trabeculectomy for patients with long-standing glaucoma or those who are not available to frequent follow-up visits. For these reasons, in our clinical practice, CP is often offered as first surgical option to elderly patients, especially if they live in distant rural areas.

The coexistence of cataract and glaucoma, which is frequently encountered in patients in this age range, poses another challenge to the surgeon, who needs to decide whether to combine both procedures and remove the lens at the same time of the canaloplasty or to proceed with two separate surgeries. The decision can be particularly challenging, especially if the cataract is not visually-significant. While a combined procedure reduces the need for additional surgery and consequent follow-up visits, there are no clear results in the literature on whether a combined phaco-canaloplasty (PCP) procedure is more effective than CP alone in reducing IOP and glaucoma medications [18–21]. Moreover, a significant proportion of our patient population is affected by pseudoexfoliative glaucoma (PEG), which has a more aggressive course than POAG and conveys a higher risk of complications after cataract surgery, such as elevated IOP spikes in the postoperative period [22, 23]. Brusini et al. have advised against performing conventional CP in patients with PEG due to the higher risk of late onset IOP spikes [24]. However, no study has evaluated the outcome of PCP in patients with PEG and cataract.

The aim of this study was to evaluate the long-term efficacy of CP in our local glaucoma population over a period of four years and to compare the surgical outcome of CP with PCP based on the postoperative reduction of IOP and number of glaucoma medications. We also evaluated which preoperative factors influence the surgical outcome of both procedures.

## Methods

### Patients and follow-up

This retrospective, single-center, longitudinal study was conducted at the Ophthalmology clinic of the University of Trieste, Italy, and included patients with OAG who underwent CP or PCP between January 2015 and January 2022. Inclusion criteria were an age ≥ 18 years, a diagnosis of OAG without prior glaucoma surgery and a follow-up > 6 months. Exclusion criteria were a diagnosis of major eye diseases other than glaucoma (such as retinal detachment or uveitis), secondary glaucoma (uveitic, post traumatic, phacogenic etc.), closure angle glaucoma, narrow angle eyes and a history of other intraocular surgeries except for cataract surgery. Surgical indications to CP were an IOP exceeding the individual target pressure on maximum tolerated medical therapy and a significant progression of the visual field defect in two consecutive visual field exams. Preoperatively, all patients underwent a complete ophthalmological evaluation, including best corrected visual acuity exam (BCVA), pupillary reflexes exam, IOP measurement by Goldmann applanation tonometry, gonioscopy angle grading according to the Shaffer system, slit lamp biomicroscopy of the anterior segment and fundus indirect ophthalmoscopy of the optic nerve head. In addition, Humphrey visual field (HVF) 24–2 exam (Humphrey Field Analyzer 3, Zeiss), central corneal thickness (CCT), axial length (AL) and keratometric parameters required for the intraocular lens (IOL) calculation (IOLMaster® 700, Zeiss) were obtained. An average of two IOP measurements taken within 1 month before the surgery was used as preoperative IOP value. The number and type of preoperative glaucoma medications entered in the analysis were obtained from the last preoperative visit. Postoperative IOP and the number and type of glaucoma medications were recorded at 6-month intervals until the patient was lost to follow-up or underwent additional procedures to control the IOP, including laser (laser trabeculoplasty, cyclophotocoagulation) or surgery (trabeculectomy or valve implant). Informed consent was obtained by each patient. The study was approved by the Ethics Committee of the University of Trieste and adhered to the tenets of the Declaration of Helsinki.

### Surgical technique

After peri-bulbar anesthesia, conjunctiva and Tenon’s capsule were dissected, creating a fornix-based flap at 12 o’clock position. A superficial triangular scleral flap of approximately one third of the total scleral thickness was dissected, carrying forward the incision up to clear cornea. A smaller and deeper scleral flap was then sculpted just above the choroidal plane and extended forward to slice the scleral spur. In this way, Schlemm’s canal was opened and a trabeculo-descemetic window created. At this point, the internal wall of Schlemm’s canal was removed with micro-surgical forceps. The deep flap was then dissected away and the two ostia of Schlemm’s canal exposed. Repeated visco-dilatation of the ostia with a microcannula was performed. The microcatheter (iTrack 250, Ellex iScience, Inc., Freemont, CA, USA) was inserted into Schlemm’s canal through the exposed ostia and advanced throughout the 360°. When the distal tip of the catheter emerged at the opposite site, a 10–0 Prolene suture was tied to the tip and the microcatheter was withdrawn, injecting approximately 0.5 ul of viscoelastic material every 2 h. The tip was then extracted from Schlemm’s canal at the initial surgical site and a 10–0 nylon suture knotted under tension to obtain an inward distension of the trabecular meshwork. Finally, the superficial scleral flap was tightly closed with four 10–0 nylon stitches to ensure a watertight closure and the conjunctival flap was sutured. In phakic patients requiring cataract extraction, phacoemulsification with intraocular lens (IOL) implantation was performed by creating the main corneal incision in the temporal sector, before canaloplasty.

### Statistical analysis

Baseline characteristics between surgical groups were compared by using unpaired two-tailed *t*-test for continuous variables such as age, cup to disc ratio (c/d), CCT, AL, visual field parameters (MD, PSD), BCVA and IOP, and by Pearson Chi-square test for categorical variables such as male/female ratio, type of glaucoma (POAG/PEG), phakic status, number of antiglaucoma medications. A fixed combination was calculated as two medications, whereas oral acetazolamide was counted as one medication. Postoperative changes in IOP and number of glaucoma medications were assessed for all the study eyes and separately, for CP and PCP eyes, by repeated-measures ANOVA. Group differences at each 6-month time point were evaluated by unpaired two-tailed *t*-test for a follow-up period of 42 months. Surgical success was classified into 6 categories according to target IOP values (≤ 21 mmHg, ≤ 18 mmHg and ≤ 15 mmHg) with or without the use of hypotensive medications (qualified and complete success, respectively) in the postoperative period. The surgery was deemed a failure if IOP was lower than 6 mmHg or whether a laser or surgical procedure was performed to reduce the IOP*.* Kaplan–Meier survival analysis and the log-rank test were used to compare the cumulative incidence of qualified and complete success between the considered groups up to 42 months. Patients who received additional laser and surgical treatment, including selective laser trabeculoplasty, cyclophotocoagulation, trabeculectomy and valve implant, were censored form further analysis and classified as failures on Kaplan–Meier analysis. Survival analysis with Cox regression was performed to identify baseline patients’ characteristics associated with postoperative outcomes. Univariable analysis was first used to identify potential prognostic factors and was further confirmed by multivariable analysis. The variables entered in the analysis were age, gender, AL, glaucoma type (POAG vs PEG), preoperative IOP, number of preoperative antiglaucoma medications and type of surgery (CP vs PCP). Kaplan–Meier survival analysis and the log-rank test were used to compare the cumulative incidence of qualified and complete success between CP and PCP eyes on different numbers of preoperative glaucoma medications. The percentages of post-operative complications between CP and PCP were compared by Pearson’s Chi-square test. P values lower than 0.05 were considered statistically significant. Data were analyzed using the IBM SPSS Statistics for Windows, Version 20.0. (IBM Corp, Armonk, NY, USA).

## Results

### Patient demographics

One-hundred ninety eyes from 170 patients were included in the study, with 28 eyes (14.7%) completing the 48-month-visit. Of the remaining 162 eyes, 110 (57.9%) belonged to patients who were lost to follow-up and 52 (27.4%) had additional glaucoma surgery or laser procedure during the follow-up period and were thereafter excluded from the analysis. Patient baseline characteristics are summarized in Table 1. One-hundred thirty-three eyes underwent CP and 57 eyes were treated with PCP. No significant differences in age, gender, glaucoma type (POAG vs PEG), C/D, visual field parameters (MD and PSD) and preoperative IOP or number of hypotensive medications were found between the two groups. The only significant different variables were CCT, which was slightly thinner in PCP eyes compared to CP eyes by 13 μm (*p* = 0.04, unpaired two-tailed *t*-test), axial length, which was significantly higher by 0.6 mm in CP eyes compared to PCP eyes (*p* = 0.001) and BCVA, which was lower in PCP patients, most likely for the presence of lens opacities in this group (*p* = 0.009).

**Table 1:** Summary of patient characteristics at baseline

	Canaloplasty	Phaco-canaloplasty	*P* value
*n*	133	57	
Age	75.71 ± 9.17	74.60 ± 7.60	*0.429*
Gender			*0.258*
*Male*	59 (*44.36%*)	28 (*49.12%*)	
*Female*	74 (*54.64%*)	29 (*50.88%*)	
Type of Glaucoma			*0.098*
*POAG*	78 (*58.64%*)	26 (*45.61%*)	
*PEG*	55 (41.36%)	31 (*53.39%*)	
C/D	0.68 ± 0.16	0.73 ± 0.13	*0.232*
CCT	534.69 ± 37.82	521.06 ± 44.89	***0.04***
AL	24.18 ± 2.00	23.48 ± 0.92	***0.001***
Phakic status			***0.000***
*Phakic*	22 (*16.54%*)	57 (*100%*)	
*Pseudophakic*	110 (*82.71%*)	–	
*Aphakic*	1 (*0.75%*)	–	
Visual field parameters			
*MD*	−11.22 ± 10.28	−14.10 ± 9.39	*0.17*
*PSD*	6.67 ± 5.53	7.85 ± 3.69	*0.495*
BCVA	0.78 ± 0.86	0.59 ± 0.29	***0.009***
IOP	21.21 ± 5.03	20.40 ± 4.11	*0.287*
Glaucoma medications	3.94 ± 1.01	3.86 ± 1.00	*0.275*
*#1*	4 (*3.01%*)	1 (*1.75%*)	
*#2*	6 (*4.51%*)	2 (*3.51%*)	
*#3*	25 (*18.80%*)	19 (*33.33%*)	
*#4*	61 (*45.86%*)	18 (*31.58%*)	
*#5*	33 (*24.81%*)	16 (*28.08%*)	
*#6*	4 (*3.01%*)	1 (*1.75%*)	

*n* = sample size, POAG = primary open angle glaucoma, PEG = pseudoexfoliative glaucoma, C/D = cup to disc ratio, CCT = central corneal thickness, AL = axial lenght, MD = mean deviation, PSD = pattern standard deviation, BCVA = best corrected visual acuity, IOP = intraocular pressure

### Postoperative changes in intraocular pressure and number of *glaucoma* medications

**Fig. 1:**
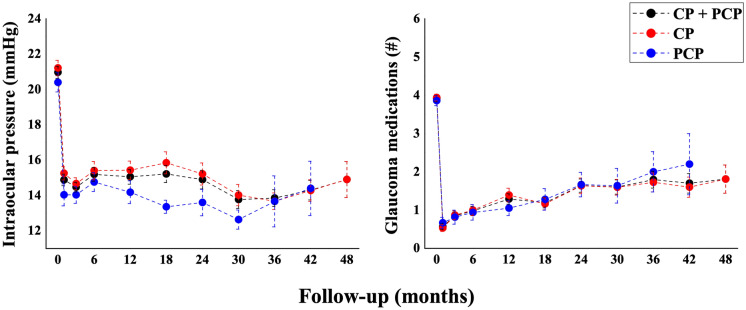
The graphs illustrate postoperative changes in IOP (on the right) and glaucoma medications (on the left) in all study eyes, treated with either canaloplasty or phaco-canaloplasty (black dots), and separately in eyes which underwent canaloplasty (red dots) and phaco-canaloplasty (blue dots) during the follow-up period of 48 months. (CP = canaloplasty, PCP = phaco-canaloplasty). Values are expressed as mean ± standard error

Figure 1 illustrates the changes in IOP and number of glaucoma medications for all the study eyes and separately for CP and PCP groups during the entire 48-month period. In all the study eyes (CP + PCP), mean IOP decreased by 31% at 48 months compared to baseline, from 21.0 ± 4.8 mmHg (Mean ± SD) to 14.5 ± 4.7 mmHg. Similarly, the number of hypotensive medications dropped from a mean preoperative value of 3.9 ± 1.0 to 1.7 ± 1.7 at 48 months. In CP patients, IOP decreased from baseline to 48 months by 30%, from 21.2 ± 5.0 to 14.9 ± 4.7 mmHg and the number of hypotensive medications decreased from a mean preoperative value of 3.9 ± 1.0 to 1.8 ± 1.7. In PCP patients, the decrease in IOP from baseline to 42 months was 29.5%, from 20.4 ± 4.1 to 14.4 ± 3.4 mmHg and the number of antiglaucoma medications decreased from a mean preoperative value of 3.9 ± 1.0 to 2.2 ± 1.8. (Table 2). For all 3 groups, repeated-measures ANOVA showed a significant reduction in both IOP and number of glaucoma medications at each time point during the postoperative follow-up compared to baseline (*p* = 0.001). PCP eyes showed on average a greater reduction in IOP compared to CP eyes, by 1 mmHg. However, no significant differences in IOP or number of glaucoma medications were found between CP and PCP groups during the follow-up, except for the 18-month time point, when IOP in PCP patients was significantly lower than in CP patients (13.36 mmHg vs 15.9 mmHg, *p* = 0.002, unpaired two-tailed *t*-test). Figure S1 shows individual postoperative changes in IOP and number of glaucoma medications for both CP and PCP groups at 6-month intervals. At each time point, most dots are localized in the right lower section of the graphs, indicating that the majority of eyes showed a decrease in both IOP and number of glaucoma medications compared to baseline. The percentages of all the study eyes with decremental postoperative changes in both IOP and number of glaucoma medications were 76% at 12 months, 80% at 24 months, 80% at 36 months and 78% at 48 months. When comparing CP and PCP groups by using this criterion, the percentages of eyes with a reduction in both IOP and glaucoma medications were 72% vs 87% (*p* = 0.045, Pearson’s Chi-square test) at 12 months, 75% vs 80% (*p* = 0.75, Fisher’s Exact Test) at 24 months, 80% vs 78% (*p* = 1.00, Fisher’s Exact Test) at 36 months, and 84% vs 60% at 42 months, (*p* = 0.25, Fisher’s Exact Test) respectively.

**Table 2:** Postoperative IOP and glaucoma medication number

Follow-up (months)	All eyes			Canaloplasty			Phaco-canaloplasty		
	n	Mean IOP ± SD (mmHg)	Mean Meds ± SD (*n*)	n	Mean IOP ± SD (mmHg)	Mean Meds ± SD (*n*)	n	Mean IOP ± SD (mmHg)	Mean Meds ± SD (*n*)
Baseline	190	21.0 ± 4.8	3.9 ± 1.0	133	21.2 ± 5.0	3.9 ± 1.0	57	20.4 ± 4.1	3.9 ± 1.0
Post-op									
3	174	14.5 ± 3.7	0.9 ± 1.3	120	14.7 ± 3.7	0.9 ± 1.3	54	14.0 ± 3.5	0.8 ± 1.4
6	151	15.2 ± 4.8	1.0 ± 1.4	101	15.4 ± 5.2	1.0 ± 1.5	50	14.8 ± 3.8	0.9 ± 1.4
12	128	15.0 ± 4.7	1.3 ± 1.6	89	15.4 ± 4.9	1.4 ± 1.7	39	14.2 ± 4.0	1.1 ± 1.2
18	98	15.2 ± 4.8	1.2 ± 1.4	73	15.9 ± 5.3	1.2 ± 1.4	25	13.4 ± 1.9	1.3 ± 1.4
24	75	14.9 ± 4.6	1.6 ± 1.5	60	15.2 ± 4.8	1.6 ± 1.6	15	13.6 ± 2.9	1.7 ± 1.2
30	62	13.8 ± 4.0	1.6 ± 1.5	51	14.0 ± 4.3	1.6 ± 1.5	11	12.6 ± 1.8	1.6 ± 1.5
36	49	13.9 ± 3.3	1.8 ± 1.5	40	13.7 ± 3.0	1.7 ± 1.6	9	13.7 ± 4.3	2.0 ± 1.8
42	30	14.3 ± 3.0	1.7 ± 1.4	25	14.3 ± 3.0	1.6 ± 1.3	5	14.4 ± 3.4	2.2 ± 1.8
48	23	14.5 ± 4.7	1.7 ± 1.7	21	14.9 ± 4.7	1.8 ± 1.7	2*	10.5 ± 2.1*	1.6 ± 1.6*

IOP = intraocular pressure, * = data not considered in the final analysis

### Surgical success

Table 3 shows the rate of surgical success for eyes undergoing either CP or PCP, stratified according 3 different values of postoperative IOP values (≤ 21 mmHg, ≤ 18 mmHg or ≤ 15 mmHg) with or without glaucoma medications (qualified vs complete success, respectively). At 48 months, 86% of CP eyes had an IOP of 18 mmHg or less with the use of medications and 33% without the use of glaucoma medications. At 42 months, 100% of PCP eyes had an IOP of 18 mmHg or less with the use of medications and 20% without the use of glaucoma medications.

**Table 3:** Success results in canaloplasty and phaco-canaloplasty groups

	Success rate (%)								
	Canaloplasty					Phaco-canaloplasty			
	12 Months	24 Months	36 Months	42 Months	48 Months	12 Months	24 Months	36 Months	42 Months
Qualified success									
IOP ≤ 21 mmHg	92	93	97	96	90	97	100	89	100
IOP ≤ 18 mmHg	87	88	92	88	86	89	93	89	100
IOP ≤ 15 mmHg	63	70	68	80	67	71	73	78	67
Complete success									
IOP ≤ 21 mmHg	51	42	32	28	33	51	20	30	20
IOP ≤ 18 mmHg	51	42	29	24	33	47	20	30	20
IOP ≤ 15 mmHg	39	35	26	24	29	37	13	20	20

IOP = intraocular pressure

**Fig. 2:**
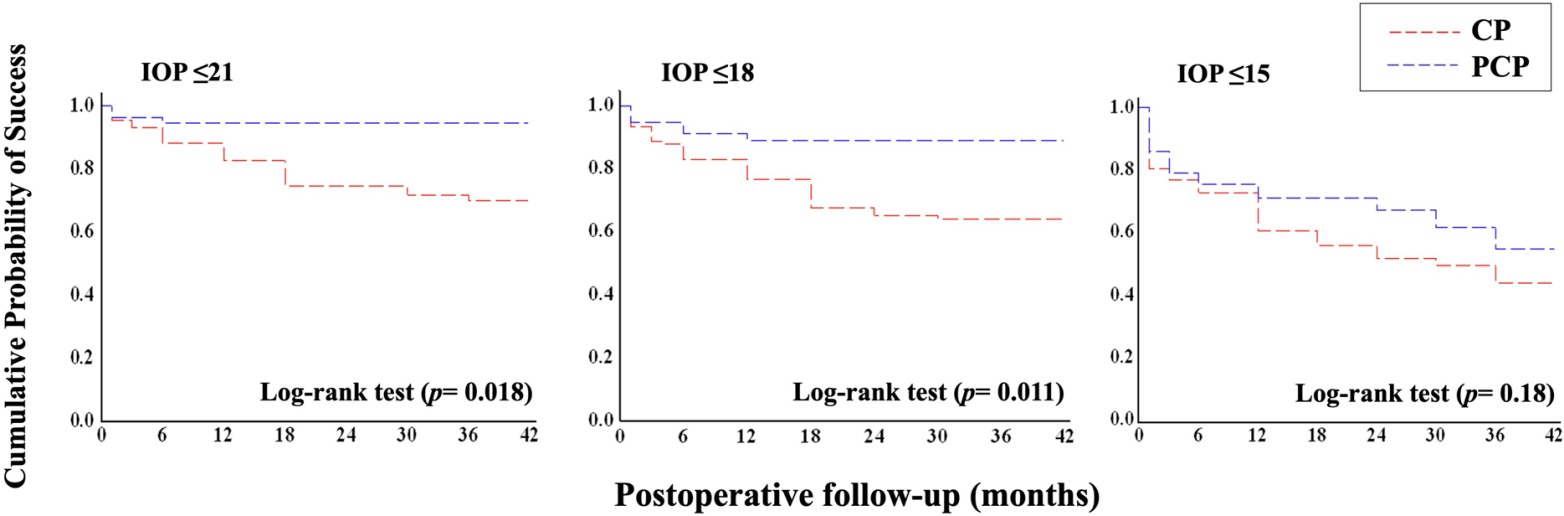
Kaplan–Meier plots of the cumulative probability of surgical success in canaloplasty and phaco-canaloplasty eyes for 3 different criteria of success. Surgical success was defined as an IOP ≤ 21, ≤ 18 and ≤ 15 mmHg. (CP = canaloplasty, PCP = phaco-canaloplasty)

Figure 2 shows the Kaplan–Meier plots in PC and PCP eyes using the survival criteria of a postoperative IOP ≤ 21 mmHg, ≤ 18 mmHg and ≤ 15 mmHg. The log-rank test showed a significant difference between CP and PCP for an IOP ≤ 21 mmHg and ≤ 18 mmHg (*p* = 0.018 and *p* = 0.011, respectively), but not for an IOP ≤ 15 mmHg, indicating that at 42 months PCP was associated with a higher probability of cumulative surgical success compared to CP, except for target IOPs below 16 mmHg. No significant differences were found between CP and PCP when considering other surgical success criteria.

### Prognostic factors

Multivariate cox regression analysis identified PCP as a significant prognostic factor over CP in achieving a qualified surgical success for post-operative IOPs ≤ 21 and ≤ 18 mmHg. A greater number of glaucoma medications at baseline was associated with a higher rate of surgical failure. The difference was significant for all the success criteria except for a qualified success with a IOP ≤ 15 mmHg, where, however, the result approached the statistical significance. Pairwise comparison of Kaplan Meier curves showed significant a higher surgical success rate in patients treated preoperatively with ≤ 4 glaucoma medications compared with those taking 5 or more medications. There were no differences in survival rate between patients taking ≤ 2 medications and those on 3 or 4 drugs (data not shown). A greater IOP was also identified as a negative prognostic factor for all the surgical success criteria, except for a complete success with a IOP ≤ 15 mmHg. Despite being statistically significant, the effect of preoperative IOP on the rate of surgical success was rather modest, as shown by the HR ranging from 1.03 to 1.10. None of the other preoperative parameters (age, gender, axial length and type of glaucoma) was significantly associated with surgical success rate (Table 4).

**Table 4:** Results of multivariable analysis using the Cox proportional hazards model

	Age			Gender			Axial length			POAG vs PEG			Pre-op IOP			Pre-op # of medications			PCP vs CP		
	HR	95% CI	*p*	HR	95% CI	*p*	HR	95% CI	*p*	HR	95% CI	*p*	HR	95% CI	*p*	HR	95% CI	*p*	HR	95% CI	*p*
Qualified success																					
IOP ≤ 21 mmHg	1.03	(0.68–1.54)	*0.90*	1.59	(0.74–3.40)	*0.24*	0.61	(0.21–1.78)	*0.37*	1.25	(0.59–2.60)	*0.56*	1.09	(1.03–1.16)	***0.01***	1.84	(1.21–2.80)	***0.00***	0.16	(0.04–0.70)	***0.01***
IOP ≤ 18 mmHg	1.15	(0.80–1.63)	*0.46*	1.08	(0.59–1.98)	*0.81*	0.46	(0.18–1.18)	*0.11*	1.16	(0.63–2.15)	*0.64*	1.10	(1.04–1.16)	***0.00***	1.43	(1.02–1.98)	***0.04***	0.37	(0.16–0.89)	***0.03***
IOP ≤ 15 mmHg	1.06	(0.81–1.38)	*0.67*	1.02	(0.66–1.60)	*0.91*	0.82	(0.44–1.50)	*0.51*	1.05	(0.66–1.65)	*0.85*	1.09	(1.04–1.13)	***0.00***	1.27	(1.00–1.61)	***0.05***	0.79	(0.47–1.33)	*0.37*
Complete success																					
IOP ≤ 21 mmHg	1.24	(0.98–1.58)	*0.74*	0.96	(0.64–1.43)	*0.83*	1.25	(0.76–2.03)	*0.38*	0.79	(0.52–1.20)	*0.27*	1.06	(1.02–1.10)	***0.00***	1.54	(1.22–1.94)	***0.00***	1.21	(0.78–1.90)	*0.38*
IOP ≤ 18 mmHg	1.28	(1.02–1.60)	***0.03***	1.06	(0.72–1.56)	*0.76*	1.10	(0.68–1.80)	*0.71*	0.89	(0.60–1.32)	*0.57*	1.05	(1.02–1.09)	***0.01***	1.53	(1.23–1.90)	***0.00***	1.18	(0.77–1.81)	*0.46*
IOP ≤ 15 mmHg	1.16	(0.93–1.45)	*0.19*	1.09	(0.75–1.60)	*0.65*	1.17	(0.74–1.87)	*0.50*	1.07	(0.73–1.56)	*0.75*	1.03	(0.99–1.07)	*0.78*	1.34	(1.10–1.64)	***0.00***	1.17	(0.77–1.78)	*0.46*

IOP = intraocular pressure, HR = hazard ratio, CI = confidence interval, POAG = primary open angle glaucoma, PEG = pseudoexfoliative glaucoma, PCP = phaco-canaloplasty, CP = canaloplasty

### Early postoperative complications

Table 5 shows the rate of postoperative complications observed within the first month after surgery in both treatment groups. Whereas the incidence of hyphema/microhyphema and hypotony (IOP < 6 mmHg) did not differ between CP and PCP eyes (25.4% vs 17.5%, *p* = 0.23 for hyphema and 5.2% vs 1.8%, *p* = 0.27 for hypotony, respectively, Pearson’s Chi-square test), PCP was complicated by a significantly higher rate of elevated IOP (> 25 mmHg) compared to CP (40.4% vs 12.7%, *p* < 0.001).

**Table 5:** Early postoperative complications (within 1 month)

	Canaloplasty (*n* = 133)	Phaco-canaloplasty (*n* = 57)	*P* value
Hyphema/Microhyphema	34 (*25.37%*)	10 (17.54*%*)	*0.230*
Ocular hypertension (> *25 *mmHg)	17 (*12.68%*)	23 (*40.35%*)	***0.000***
Ocular hypotony (< *7 *mmHg)	7 (*5.22%*)	1 (*1.75%*)	*0.266*

## Discussion

The aim of this single center, retrospective study was to assess the long-term efficacy of CP and PCP in our local glaucoma population and to compare the outcomes of these two procedures. The results of this study show that both procedures maintain their efficacy in the long term, with CP showing efficacy up to 48 months and PCP up to 42 months, as demonstrated by a consistent and significant reduction in IOP and number of antiglaucoma medications with respect to the preoperative values.

### Postoperative efficacy of canaloplasty at 3 and 4 years

Table 6 provides a summary of previous studies on CP and PCP that utilized our same surgical technique, referred to as traditional CP, with a postoperative follow-up of at least 3 years. Except for two studies conducted by Brusini et al. with follow-ups of 48 and 72 months [24, 25], all the other studies had a follow-up of 3 years [18–21, 26]. Although it is challenging to compare studies with different patient populations and designs, our 3-year results in patients treated with CP are overall consistent with what previously reported in the literature and confirm the satisfactory antihypertensive efficacy of CP.

**Table 6:** List of studies on “ab-externo” canaloplasty and phaco-canaloplasty with a follow-up of at least 3 years

Study	Surgery	Eyes	Age (years)	Glaucoma type	Follow-up (*months*)	Baseline		Post-op changes		Surgical success rates	Early complications (< 90 days)
						IOP (*mmHg*)	Med (*n*)	IOP (*mmHg*)	Med (*n*)		
*Grieshaber *et al*. 2010* (single center, retrospective)	CP	*n* = 60	49.8 ± 15.7	POAG	30.6 ± 8.4	45.0 ± 12.1	2	13.3 ± 1.7 (↓70%)	0 (↓2.0)**	IOP ≤ 21 = 82% (QS), 78% (CS). IOP ≤ 18 = 68% CS). IOP ≤ 16 = 47%(CS).	MH (70%), IOP ≥ 30 (1.6%), DMD (3.3%)
*Bull *et al*. 2011*(multicenter, prospective)	CP	*n* = 93	67.3 ± 9.9	POAG, PEG, Pigmentary, Mixed mechanism	36	23.0 ± 4.3	1.9 ± 0.7	15.1 ± 3.1 (↓34%)	0.9 ± 0.9 (↓1.0)	IOP ≤ 21 = 99% (QS), 41% (CS). IOP ≤ 18 = 82% (QS), 37% (CS). IOP ≤ 15 = 57% (QS), 22% (CS).	MH (12.8%), H (5.5%), IOP ≥ 30 (5.5%), DMD (3.7%)
	PCP	*n* = 16				24.3 ± 6.0	1.5 ± 1.2	13.8 ± 3.2 (↓43%)	0.5 ± 0.7 (↓1.0)	IOP ≤ 21 = 100% (QS), 62% (CS). IOP ≤ 18 = 100% (QS), 62% (CS). IOP ≤ 15 = 69% (QS), 31% (CS).	
*Lewis *et al*. 2011*(multicenter, prospective)	CP	*n* = 121	67.6 ± 11.6	POAG, PEG, Pigmentary, Mixed mechanism	36	23.5 ± 4.5	1.9 ± 0.8	15.5 ± 3.5 (↓34%)	0.9 ± 0.9	IOP ≤ 21 = 95.5% (QS), 40% (CS). IOP ≤ 18 = 77.5% (QS), 36% (CS). IOP ≤ 15 = 55% (QS), 21% (CS).	MH (12.1%), H (10.2%), IOP ≥ 30 mmHg (6.4%), DMD (3.2%), IOP < 6 mmHg (0.6%)
	PCP	*n* = 36				23.5 ± 5.2	1.95 ± 1.0	13.6 ± 3.6 (↓42%)	0.3 ± 0.5	IOP ≤ 21 = 100% (QS), 78% (CS). IOP ≤ 18 = 89% (QS), 70% (CS). IOP ≤ 15 = 70% (QS), 52% (CS)	
*Tetz *et al*. 2015*(multicenter, retrospective)	CP	*n* = 82 phakic	63.5 ± 9.9	POAG, PEG, Pigmentary	36	23.4 ± 4.3	1.9 ± 0.8	15.5 ± 3.5 (↓33%)	0.9 ± 1 (↓1.0)	Not available	H (21.4%), CE (1.9%), wound leakage (7.8%), IOP > 5 mmHg than baseline (3.9%), DMD (1.9%), IOP < 5 mmHg + shallow anteriore chamber (1%)
		*n* = 21 pseudophakic	76.3 ± 8.4			23.9 ± 5.2	1.8 ± 0.8	15.6 ± 3.5 (↓35%)	1.1 ± 0.8 (↓0.7)		
	PCP	*n* = 30	74.8 ± 9			23.5 ± 5.3	1.5 ± 1.0	13.6 ± 3.6 (↓42%)	0.3 ± 0.5 (↓1.2)		H (23.3%), CE (46.7%), IOP > 5 mmHg than baseline (3.3%), DMD (3.3%)
*Khaimi *et al*. 2017*(single center, retrospective)	CP	*n* = 150	72.8 ± 10.9	POAG, PEG, Pigmentary	36	21.1 ± 7.2	2.2 ± 1.3	15.0 ± 4.6 (↓28%)	0.5 ± 0.8 (↓1.7)	IOP ≤ 21 = 95% (QS), 65%(CS). IOP ≤ 18 = 78% (QS), 59%(CS). IOP ≤ 15 = 68% (QS), 57%(CS)	H (53%)
	PCP	*n* = 127				18.1 ± 5.6	2.0 ± 1.1	15.4 ± 4.0 (↓15%)	0.7 ± 1.1 (↓1.3)	IOP ≤ 21 = 78% (QS), 48% (CS). IOP ≤ 18 = 70% (QS), 44% (CS). IOP ≤ 15 = 59% (QS), 33% (CS)	
*Brusini *et al*. 2014* (single center, retrospective)	CP	*n* = 214	63.5 ± 14	POAG, PEG, Pigmentary, Juvenile	48	29.4 ± 7.9	3.3 ± 0.9	17.0 ± 4.2 (↓42%)	1.3 ±1.5 (↓2.0)**	3 years: IOP ≤ 21 = 86% (QS), 45% (CS). IOP ≤ 18 = 59% (QS), 31% (CS). IOP ≤ 16 = 38% (QS), 24% (CS).	H (21.9%), aqueous leakage from the conjuntival flap (0.9%), IOP < 5 mmHg (9.8%), IOP spikes > 10 mmHg (5.6%), DMD (5.1%)
*Brusini *et al*. 2022* (single center, retrospective)	CP	*n* = 67	67.8 ± 12.5	PEG	49 ± 32.3	31.2 ± 8.7	3.5 ± 0.9	17.2 ± 6.7 (↓45%)*	1.9 ± 1.3 (↓1.6)***	4 years: IOP ≤ 21 = 83% (QS), 21% (CS). IOP ≤ 18 = 69% (QS), 21% (CS). IO*P* ≤ 16 = 55% (QS), 21% (CS).	H (34.1%), IOP < 5 mmHg (2.4%), DMD (4.9%), IOP spikes > 10 mmHg (9.7%)

IOP = Intraocular pressure, Med = glaucoma medications, CP = canaloplasty, PCP = phaco-canaloplasty, POAG = primary open angle glaucoma, PEG = pseudoexfoliative glaucoma, QS = qualified success, CS = complete success, H = hyphema, MH = microhyphema, DMD = Descemet's membrane detachment, CE = corneal edema, * = 24 months, ** = 36 months, *** = 48 months

Three years after CP, our patients showed a mean decrease in IOP by 35%, which was similar to what reported by the majority of 3-years studies, in which the average decrease in IOP ranged from 28 and 35% [18–21]. While these studies showed a 3-year mean IOP ranging from 15 to 15.5 mmHg, our patients showed a mean IOP of 13.7 ± 3 mmHg. This difference in IOP could be due to our population being on a higher number of glaucoma medications at 3 years i.e., 1.73 ± 1.57, compared with other studies, in which the mean number of medications ranged from 0.5 to 1.1.

Our study demonstrated a high rate of qualified success, with 88% of patients achieving an IOP ≤ 18 mmHg at 3 years. This is slightly higher than what reported in other studies, in which the rate of qualified success ranged from 53.1% to 82.4% [18, 19, 21]. However, our rate of complete success was lower, with only 24% of patients achieving an IOP ≤ 18 mmHg without glaucoma medications, while other studies reported rates between 31 and 59%. One possible explanation for this difference could be that our patients were preoperatively on a higher number of glaucoma medications (3.9 ± 1.0) than those in other studies, in which the mean number of medications ranged from 1.8 to 2.2. Therefore, although our patients achieved similar reductions in both IOP and medication numbers 3 years after CP when compared with other studies, it is possible that due to lower required IOP target levels, our patients may have necessitated a higher number of medications before, as well after, the surgery.

At 4 years, CP maintained its efficacy in controlling IOP in our glaucoma population. Although mean IOP increased to 14.9 ± 4.7 mmHg, which was on average 1.2 mmHg higher than what we reported at 3 years, IOP was still significantly reduced by 30% compared to the preoperative values. Similarly, the mean number of medications was still reduced by 2.1 units from baseline, confirming the consistent and positive effects of canaloplasty in reducing not only the IOP, but also the burden of glaucoma medications, with their associated side effects and costs. In his study on 185 patients treated with CP and followed for 4 years, Brusini et al. reported a mean IOP of 17.0 ± 4.2 mmHg and a reduction of 42% in IOP compared to baseline at 4 years. Although the authors did not provide any data on the number of medications used at 4 years, the higher IOP value and the larger reduction in IOP in their population, compared to our study, may be secondary to differences in patient selection and higher target IOPs in their patients [25].

### Phaco-canaloplasty exhibited moderately higher levels of efficacy, despite a higher prevalence of early postoperative complications compared to canaloplasty

Our study showed that PCP is not inferior to CP in achieving a long-term and steady reduction in IOP and number of glaucoma medications. At 42 months after PCP, mean IOP was 14.4 ± 3.4 mmHg and was decreased by 30% with respect to baseline, while mean number of antiglaucoma medications was 2.2 ± 1.8 and was reduced on average by 1.7 units compared with baseline. In PCP patients, IOP was reduced on average by 1 mmHg compared to CP during the all follow-up period but the difference was not statistically significant. Similarly, the number of postoperative glaucoma medications did not differ between CP and PCP during the entire follow-up.

Previous studies have provided inconsistent results when comparing the efficacy of PCP with CP. Bull et al. and Lewis et al. found higher rates of qualified and complete success in patients undergoing PCP compared to those treated with CP at 3 years [18, 19]. Similar findings were reported by Tezt et al. who found a higher percentage of patients maintaining an IOP < 18 mmHg three years after PCP [20]. Conversely, Khaimi et al. reported lower rates of surgical success in PCP patients compared to CP patients [21].

We found that PCP was associated with a higher probability of surgical success compared to CP, indicating that the removal of the crystalline lens exerted an additional effect in controlling IOP. However, the synergistic effect of canaloplasty and cataract removal was modest, as it only increased the chance of reaching a target IOP ≤ 18 mmHg on glaucoma medications. When more stringent criteria of surgical success were considered, such as a qualified success on lower target pressures i.e., an IOP ≤ 15 mmHg or a complete success for any target IOP, PCP did not prove to be more effective than CP. One possible reason for the limited effect of PCP on IOP reduction could be related to our preoperative patient selection. Pre-operative angle configuration is considered as one of the main factors contributing to the amount of IOP changes after cataract removal, with greater IOP reductions observed in patients with partially or completely closed angles [27, 28]. Because we only included patients with a wide-open angle and a deep anterior chamber, it is likely that the lens removal resulted in minimal changes to the angle anatomy after the surgery, and therefore in marginal IOP reduction.

Early postoperative complications in our population included hyphema/microhyphema and transient episodes of hypotony or elevated ocular pressure (> 25 mmHg), with a prevalence that was within the range of what reported by previous studies. Unlikely the majority of studies, we did not observe any case of Descemet’s membrane detachment. Patients who underwent PCP had a higher occurrence of elevated IOP in the first days after surgery compared to CP. This was likely secondary to the retention of viscoelastic material in the anterior chamber, to a hindered Schlemm’s canal preventing aqueous humor outflow and to higher levels of intraocular inflammation [29]. It is important to note that none of these early complications were associated with a higher risk of late surgical failure in either CP or PCP groups.

### The preoperative number of *glaucoma* medications predicts the surgical outcome

Our study identified a higher number of preoperative glaucoma medications as a significant risk factor for surgical failure. Patients taking 5 or more medications had a higher risk of surgical failure compared to those on 4 or fewer medications, ranging from 34 to 84%, depending on the criteria used to define the surgical success. We found that being affected by PEG did not influence the surgical outcomes of either CP or PCP. Brusini et al. reported that 2 to 4 years after CP, patients with PEG are at high risk of experiencing IOP spikes up to 50 mmHg, likely caused by the accumulation of pseudoexfoliative material in the angle and scarring from the prolene suture inside the Schlemm’s canal. Based on their findings, the authors recommended avoiding CP in patients with PEG [24]. Except for the early postoperative period, when both PEG and POAG eyes experienced transient hypertensive episodes as a result of the recent surgery, during our 4-year follow-up we did not observe any significant IOP spike in PEG eyes, and both PEG and POAG patients achieved comparable reductions in postoperative IOP, number of medications and surgical success rate. The efficacy of CP in PEG patients was also confirmed by a 4-year study conducted by Seuthe et al. [30]. Although the authors utilized a modified canaloplasty technique with additional suprachoroidal drainage, which may have mitigated the late increase in IOP, they achieved very successful outcomes. This was demostrated by a 45.1% reduction in IOP after canaloplasty and a 47.6% reduction in IOP after PCP at 4 years.

### Will novel modified canaloplasty techniques replace conventional canaloplasty?

The results reported in this study were obtained by using a traditional CP technique with suture placement. Over the past years various modifications of CP have been described, including catheter-less suture placement [31], intubation without viscodilatation with Glaucolight [32], CP with suprachoroidal drainage [33], or other less invasive variants such as minicanaloplasty [34] and ab-interno CP [35], each one with its advantages and limitations (see Beres & Scharioth for a recent review) [36]. A recent pilot prospective study compared three variants of CP, i.e., ab-externo, minicanaloplasty and ab-interno, combined with cataract surgery, and showed no significant differences between the 3 techniques in terms of IOP reduction, surgical success and safety profile at 12 months [37]. However, additional data and a longer follow-up are needed to confirm whether these variants can be a valid alternative to traditional CP.

## Conclusions

In conclusion, this study provides additional evidence that both CP and PCP are safe and efficient techniques for achieving a steady 30% reduction of IOP in patients with POAG and PEG, up to 48 months for CP and 42 months for PCP. PCP was moderately superior to CP in achieving a better IOP control, but this effect was lost for target IOPs below 16 mmHg, suggesting that PCP may be a potential alternative to canaloplasty in patients with mild glaucoma and cataract. It is worth noting that PCP was associated with a higher rate of complications in the early postoperative period, emphasizing the need for a closer follow-up during the initial months from the surgery in patients undergoing a combined procedure. Patients on more than 4 preoperative glaucoma medications are at higher risk of surgical failure after CP and PCP.Therefore other surgical alternatives, such as filtrating surgeries, may be more appropriate for this group of patients. The strengths of this study include the long follow-up duration, the large sample size and a single surgeon performing all the procedures. The main limitations are the retrospective design and the inclusion of patients at various stages of glaucomatous disease with different target IOPs, which may have introduced some biases in assessing the rate of surgical success in the postoperative period and, therefore, limit the generalizability of this study results.

## Supplementary Information

Below is the link to the electronic supplementary material.

Supplementary file1 (PDF 1516 kb)
