# Supplementary material for: Long-term outcomes of canaloplasty and phaco-canaloplasty in the treatment of open angle glaucoma: a single-surgeon experience
Source: Int Ophthalmol. 2024 Jul 7;44(1):317. doi: 10.1007/s10792-024-03174-x (PMC11228002; doi:10.1007/s10792-024-03174-x)
Supplement: Supplementary file 1 — Supplementary file1 (PDF 1516 kb) [file 10792_2024_3174_MOESM1_ESM.pdf]

Supplementary information (SI)

Long-term outcomes of canaloplasty and phaco-canaloplasty in the treatment of open angle glaucoma: a single-surgeon experience.

International Ophthalmology

Daniele Tognetto M.D.[1], Gabriella Cirigliano M.D.[1], Stefano Gouigoux M.D.[1], Alberto Grotto M.D.[1], Pier Luigi Guerin M.D.[1], Leandro Inferrera M.D.[1], Dario Marangoni M.D., Ph.D.[1*]

[1] University Eye Clinic, Department of Medicine, Surgery and Health Sciences, University of Trieste, 34129, Trieste, Italy. *Corresponding author.

Corresponding author:

Dario Marangoni
University Eye Clinic, Department of Medicine, Surgery and Health Sciences
University of Trieste
Piazza dell'Ospitale 1, 34129, Trieste, Italy
dario.marangoni@units.it

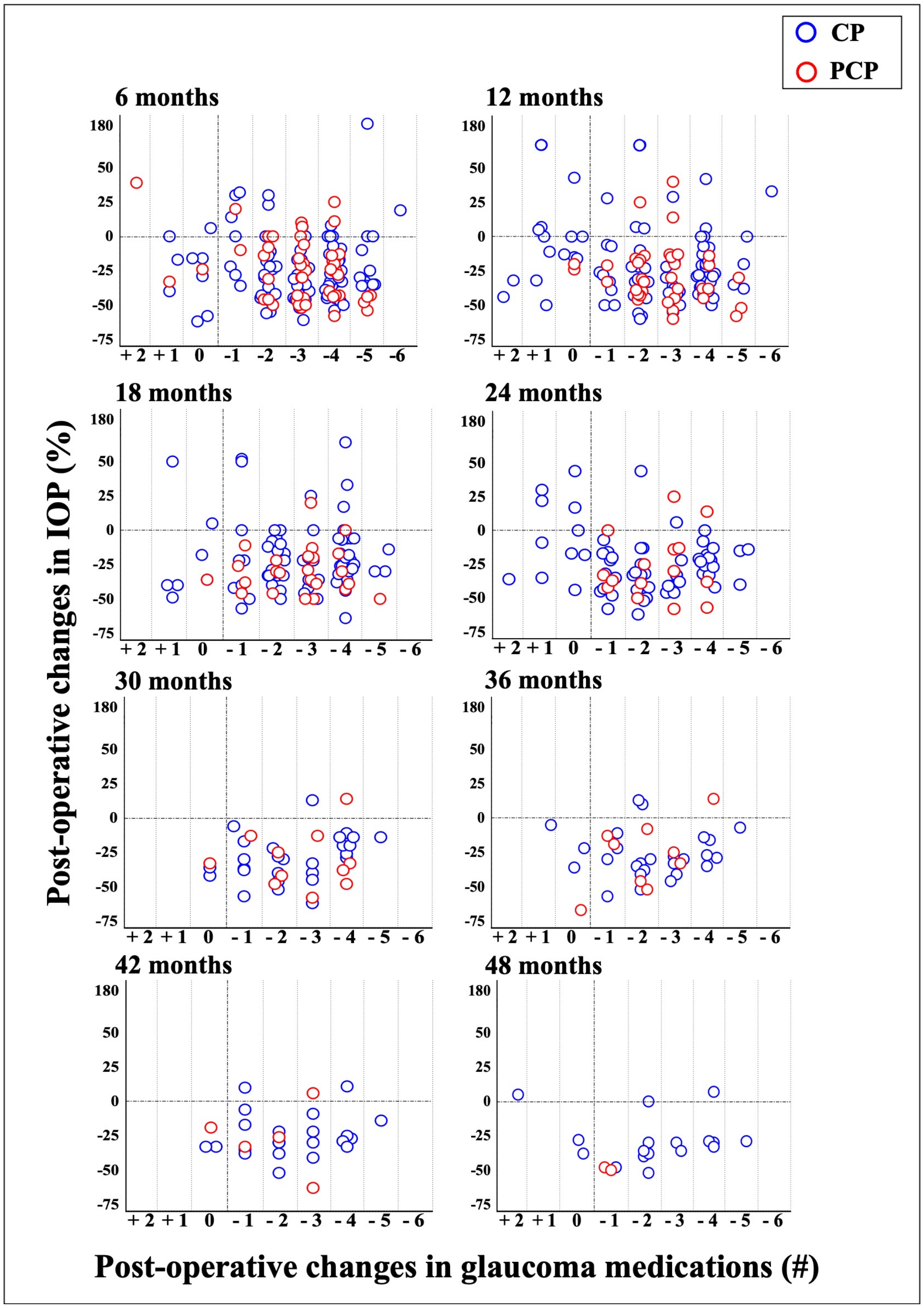

Figure S1.

The graphs illustrate individual changes in postoperative IOP and number of glaucoma medications after canaloplasty (blue dots) and phaco-canaloplasty (red dots) over a 48-month period, with data collected at six-month intervals. In each graph the lower right quadrant includes eyes in which both the IOP and number of glaucoma medications were reduced compared to baseline measurements. **CP** = canaloplasty, **PCP** = phaco-canaloplasty.
